# Perceived stress in patients with inflammatory and non‐inflammatory skin conditions. An observational controlled study among 255 Norwegian dermatological outpatients

**DOI:** 10.1002/ski2.162

**Published:** 2022-08-31

**Authors:** Flora Balieva, Christina Schut, Jörg Kupfer, Lars Lien, Laurent Misery, Francesca Sampogna, Love von Euler, Florence J. Dalgard

**Affiliations:** ^1^ Department of Dermatology Stavanger University Hospital Stavanger Norway; ^2^ Department of Public Health, Faculty of Health Sciences University of Stavanger Stavanger Norway; ^3^ Institute of Medical Psychology Justus‐Liebig‐University Gießen Giessen Germany; ^4^ Innlandet Hospital Trust Norwegian National Advisory Unit on Concurrent Substance Abuse and Mental Health Disorders Brumunddal Norway; ^5^ Innland Norway University of Applied Sciences Faculty of Social and Health Sciences Elverum Hedmark Norway; ^6^ Department of Dermatology Brest University and Regional Hospital Centre Brest Bretagne France; ^7^ Istituto Dermopatico Dell'Immacolata IDI‐IRCCS Clinical Epidemiology Unit Rome Italy; ^8^ Hudläkartjänst Stockholm Sweden; ^9^ Division of Mental Health and Addiction Vestfold Hospital Trust Tønsberg Norway; ^10^ Department of Dermatology Skåne University Hospital Lund Lund Sweden

## Abstract

**Background:**

Inflammation may increase stress, while stress may promote inflammation. Most dermatological conditions are chronic and inflammatory, while some, such as cancer, naevi and tumours are non‐inflammatory, but may cause stress because of the fear of malignancy and the necessity for surgical and other invasive treatments. Stress among patients with skin diseases is little explored.

**Objectives:**

To assess perceived stress in patients with inflammatory and non‐inflammatory skin conditions compared to healthy controls.

**Methods:**

Observational cross‐sectional study. Consecutive outpatients (*N* = 255) visiting the Department of Dermatology, Stavanger University Hospital, Norway and 148 skin‐healthy controls contributed by answering questionnaires on sociodemographics, stressful life events, economic difficulties, self‐rated health and perceived stress. The validated Perceived Stress Scale10 was used to evaluate stress. A dermatologist examined patients and registered their diagnoses and comorbidities. Controls included in this study were not examined by a dermatologist and self‐reported their comorbidities.

**Results:**

Patients with an inflammatory skin disease or psoriasis have a tripled risk of reporting moderate to high stress compared with controls when adjusted for relevant confounders, including having experienced a stressful life event recently or having a comorbidity. Patients with a purely non‐inflammatory skin disease perceived stress no differently than controls.

**Conclusion:**

Patients with inflammatory skin disease perceived higher stress than controls and patients with non‐inflammatory skin conditions. Dermatologists may play a role in awareness of the importance of stress in skin disease.

**What is already known about this topic?**

- Chronic inflammation and stress are associated. Most skin conditions are chronic and inflammatory and studies have shown the association between stress and several skin diseases, such as psoriasis, eczemas, acne and vitiligo. This association may be bidirectional where stress may exacerbate the skin disease, while increased inflammation with more symptoms from the skin may lead to further stress in a self‐perpetuating manner.

**What does this study add?**

- Our study showed a three‐fold increased perceived stress in patients with inflammatory skin diseases compared with controls when adjusted for confounders and comorbidities.
- Non‐inflammatory skin conditions (non‐melanoma skin cancer (NMSC), melanoma, tumours, actinic keratosis (AK)) were examined separately since other factors than inflammation may be the cause of stress (worse prognosis, fear of malignancy, visibility). There was no difference in perceived stress between controls and patients with purely non‐inflammatory skin disease.

## 1 INTRODUCTION

Stress is defined as any type of change that causes physical, emotional, or psychological strain.^1^ A disease with an inflammatory component, may cause or worsen stress, while stress may worsen or trigger inflammation. Other factors, such as fewer opportunities for education, employment, income and personal relationships related to a disease may contribute to lowering health related quality of life (HRQoL) and further increase stress.^2^, ^3^, ^4^

Already in 1945 Wittkover investigated the relationship between stress and psoriasis,^5^ stress is regularly reported to play an important role in the onset or exacerbation of psoriasis.^6^ Later, other inflammatory skin diseases were investigated as to their association to stress.^7^, ^8^, ^9^, ^10^, ^11^ By the late 1980s, instruments for measuring stress had been validated,^12^ marking the beginning of robust and valid studies on stress.^12^

During the last 2 decades multiple studies have shown that patients with skin disease have a lower HRQoL,^4^, ^13^ higher risk for depression, anxiety,^14^ stress^15^ and issues with body image.^4^, ^16^, ^17^ Perceived stress is less explored, but low HRQoL, depression and anxiety related to the skin condition can increase stress, which in turn may worsen the skin disease, reduce the HRQoL even more, resulting in a self‐perpetuating process.^3^, ^4^, ^15^, ^18^

Sensitivity to stress affects psoriasis severity and high stress reactors had significantly more severe psoriasis, where stress, in turn, exacerbated the skin condition.^6^ Other studies conclude that psychological stressors can contribute to the first manifestation and exacerbations of psoriasis.^19^, ^20^, ^21^ We have evidence to support the anecdotal patient reports that stress worsens psoriasis^6^, ^15^, ^22^, ^23^ and other inflammatory skin diseases.^7^, ^8^, ^9^, ^11^, ^15^, ^24^, ^25^, ^26^ A case controlled study reported that stressful events were associated with the recurrence and exacerbation of psoriasis.^27^

Studies on stress and skin disease exist for psoriasis, atopic dermatitis, hand eczema, urticaria, acne^9^, ^25^ and vitiligo,^8^, ^9^, ^28^ and show results in favour of inflammatory diseases perpetuating stress.^27^ However such studies lack for many other dermatological conditions.

Pathoethiological mechanisms in stress have been explored and stress is used to explain medical problems,^29^ while also considered a risk factor for morbidity. However, ‘stress’ is not a diagnostic entity, therefore no patient registers exist to be able to analyse associations between stress and inflammatory disease.

Biological mechanisms for stress and skin disease involve the neuro‐immuno‐cutaneous system and hypothalamic‐pituitary‐endocrine axis, where the release of neuropeptides, catecholamines and cortisol is increased. The skin produces neuropeptides such as corticotropin‐releasing hormone, substance *P* or calcitonin gene‐related peptides in response to stress. Neurotransmitters can modulate skin cell properties. Inflammatory cells are most sensitive to neurotransmitters^3^, ^18^ and inflammatory dermatoses are believed to be more rapidly and more efficiently modulated by stress.^9^ A recent study by Jusuf et al.^26^ confirmed the release of substance *P* in acne patients, but not in controls, while another study showed serum neurotrophins to be higher in lichen simplex compared with controls.^30^

One study showed that patients with pigmented tumours experienced significantly lower stress compared with other skin conditions.^9^ However, stress associated with non‐inflammatory skin conditions is not previously explored, and the abovementioned biological mechanisms would not apply if no inflammation is present.^9^, ^31^ However, tumours, even when benign may cause concerns in the patient, such as, the fear of malignancy before the diagnosis is confirmed. It can be assumed that receiving a diagnosis of a serious skin condition, even if not inflammatory (malignant melanoma, skin cancer), may be perceived as a stressful life event initiating a state of chronic stress. Treatments, such as surgical removal and side‐effects of topical anti‐cancer medications may be more burdensome than the tumour itself.^32^ A study on a large sample of European outpatients showed that therapies used for cancers are burdensome and the reason for the reduced HRQoL.^32^

Whether a non‐inflammatory skin condition, due to anxiety for malignancy, can cause stress is not previously explored. Congenital skin malformations may be benign, without inflammation, yet permanent, and without treatment options, leading to stigmatisation and high experienced stress.^4^, ^16^ Non‐inflammatory lesions (benign tumours, naevi and AK) may cause more stress purely because of the patient's perceived seriousness of the lesion.

The aim of this study was to assess the association between skin disease and perceived stress and more specifically to compare stress in patients with inflammatory and non‐inflammatory skin conditions with controls.

Our secondary aim was to explore if patients with non‐inflammatory skin conditions showed high perceived stress due to the seriousness of the diagnosis or burden of treatment.

## 2 PATIENTS AND METHODS

This is a cross‐sectional, observational, single‐centre study conducted at Stavanger University Hospital (ID702), approved by the Norwegian Regional Ethical Committee 2017/1824 and is part of a larger European study, of which the protocol and first data have been published previously.^16^, ^17^ The study was conducted in concordance with the Declaration of Helsinki.

Members of the Norwegian dermatological patient society were asked to comment on the design and research questions, and their opinion was considered when planning the study.

### 2.1 Participants

Patients were recruited at the Department of Dermatology, Stavanger University Hospital, Norway between 22.03.18 and 26.11.18. Skin‐healthy controls were recruited between 19.04.18 and 10.01.19. Consecutive outpatients, not selected for any diagnosis, were approached at arrival and informed of the study. Controls (actively working and with no skin disease)^33^ were recruited from non‐clinical hospital staff.^16^ All were adult individuals (age≥18 years), able to read and understand the questions and give consent before inclusion.

Participants provided written informed consent and were free to withdraw at any point without a reason.

### 2.2 Measures

*Sociodemographic variables* were self‐reported, age (in years), sex (male or female), level of education (possibility to go to college/university or not), marital status (single or living with a partner/married), household income (low, middle, high), serious economic difficulties (yes/no).

*Some health related questions* were likewise self‐reported. These were weight and height for calculating body mass index (BMI, weight kg/height cm^2^), the occurrence of stressful life events by the question “Have you had any stressful life events during the last 6 months (serious illness, death of a close friend or a family member, accident, divorce or other events)?” (yes/no).

*Clinical assessment* was performed by the dermatologist, who classified the skin condition according to ICD‐10 criteria (International Classification of Diseases, 10th Revision). If a patient had more than one dermatological condition, no more than two were registered. The dermatologist recorded comorbidities (cardiovascular disease, chronic respiratory disease, diabetes, rheumatological disease or other). Patients answered questions regarding their skin condition. Controls were not examined clinically and self‐reported their comorbidities.

#### 2.2.1 *Psychological variables* were assessed by validated questionnaires

Depression and anxiety were measured by the Patient Health Questionnaire (PHQ‐2) and the General Anxiety Disorder Assessment (GAD‐2).^34^ Values of three or higher indicate that the individual needs to be screened for depression or anxiety. Participants with missing values were not included in the analysis.

Participants rated their health state on a visual analogue scale from zero (worst imaginable) to 100 (best imaginable), using the EuroQol‐five dimension‐Visual Analogue scale (EQ‐VAS).^35^

Perceived stress was measured with the Perceived Stress Scale (PSS‐10), a 10‐item scale that evaluates the degree to which an individual has perceived life as unpredictable, uncontrollable and overloading, if external demands exceed the individual's perceived ability to cope during the previous month.^12^, ^36^ Contrary to other scores which measure stressful life events that had occurred, PSS measures the degree to which situations in one's life are appraised as stressful.^12^, ^36^, ^37^ One dimension is related to perceived stress, another to coping ability and stress resilience.^29^ In our study we used a simplistic approach and were interested in the general perceived stress using cut‐offs as described by Cohen et al.^12^ and also used in other studies,^10^ where values are considered: ‘experiencing low stress’ (0–13), ‘moderate stress’ (14–26) and ‘high stress’ (27–40). Individuals who did not give valid responses to all questions were excluded from calculations on perceived stress.

Extensive information on these instruments is given in the study protocol.^16^

### 2.3 Data collection and statistical analyses

Following data collection, dermatological conditions were grouped by four experienced dermatologists (Flora Balieva, Anthony Bewley, Florence Dalgard, Uwe Gieler). Data were systematically checked for mistakes and statistically analysed using International Business Machines Statistical Package for the Social Sciences 26^®^. The rationale for grouping diseases is described in detail elsewhere.^17^

### 2.4 Diagnostic categories

In the Norwegian sample of 255 patients the distribution of diagnoses was wide, some diagnoses modestly represented. For the purpose of the study we pooled clinically relevant diagnoses into inflammatory and non‐inflammatory conditions.

Some patients had an inflammatory and a non‐inflammatory skin condition simultaneously. To avoid the influence of inflammation we only assigned patients to the non‐inflammatory group if they did not concurrently have an inflammatory skin disease. Patients with no primary or secondary inflammatory skin condition (*N* = 82) were considered as having a purely non‐inflammatory skin condition. This allowed us to answer our objective on whether non‐inflammatory disease via mechanisms other than inflammation may lead to higher perceived stress.

Within the inflammatory skin conditions (*N* = 173) we also present data for psoriasis, regardless if the diagnosis was registered as primary or secondary (*N* = 54). Psoriasis is a disease with high prevalence in Norway,^38^ associated with systemic inflammation^39^, ^40^ and is the most studied skin disease so far.

Supplementary Table 1, shows distribution of diagnoses and the groups they are assigned to.

### 2.5 Statistics

Categorical variables are presented by numbers and percentages, continuous variables by means and standard deviation. Dermatological patients were compared with controls regarding sociodemographic variables, self‐reported health outcomes and psychological factors using *t*‐tests for continuous variables and χ^2^‐tests for categorical variables. Only *p* values < 0.01 were considered significant to avoid a type I error when calculating results for four groups. To determine the risk of dermatological patients having higher perceived stress compared with controls, adjusted Odds Ratio (OR) and 95% confidence intervals (95% CI) were determined.

For the purpose of logistic regression, PSS was dichotomised into low perceived stress (PSS ≤ 13) and moderate to high perceived stress (PSS > 13). Stepwise binary logistic regression analyses were conducted using PSS‐10 as the criterion variable (cut‐off PSS > 13 as moderate to high stress) to determine whether perceived stress could be predicted by sociodemographic factors and health related outcomes. Age, sex, the presence of at least one comorbidity and a recently experienced significant stressful life event were included in the models as possible confounders.

## 3 RESULTS

### 3.1 Sample characteristics

There was a high participation rate of 91.7%. The total number of participants was 403 (255 patients and 148 controls). The mean age (SD) of patients was 50.9 (18.5) and of controls 40.1 (13.3) years. In both groups there were more females than males (approximately male to female 1:2, regardless of diagnostic group). Details on participant characteristics for patients, controls and diagnostic category are given in Table 1.

**TABLE 1:** Patient and control characteristics

Population	Controls *N* = 148	All patients *N* = 255	Inflammatory *N* = 173	Psoriasis *N* = 54	Non‐inflam‐matory^a^ *N* = 82
Age mean (SD)	40.1 (13.3) missing: 0	**50.9 (18.5%)** missing: 0	**45.4 (16.9%)** missing: 0	**47.2 (13.9%)** missing: 0	**62.4 (16.2)** missing: 0
Sex male *N* (%)	44 (29.7%)	80 (31.4%)	56 (32.4%)	20 (37%)	24 (29.3%)
Sex female *N* (%)	104 (70.3%)	175 (68.6)	117 (67.6%)	34 (63%)	58 (70.7%)
BMI mean (SD)	23.9 (3.7) missing: 3	**26.2 (4.9)** missing: 11	**26.7 (5.2)** missing: 8	**27.9 (5.7)** missing: 1	25.2 (4.1%) missing: 3
Education, college or university (yes)	144 (98.6%) missing: 2	**189 (79.7%)** missing: 18	**134 (81.7%)** missing: 9	**4 (83%)** missing: 1	55 (75.3%) missing: 9
Study or work (yes)	146 (98.6%) missing: 0	**143 (56.3%)** missing: 1	**109 (63.4%)** missing: 1	**35 (64.8)** missing: 0	34 (41.5) missing: 0
Marital status (yes) (married or partner)	93 (63.7%) missing: 2	166 (67.5%) missing: 9	106 (63.9%) missing: 7	32 (60.4%) missing: 1	60 (75%) missing: 2
Economic difficulties (yes)	6 (4.1) missing: 1	26 (10.4%) missing: 4	**23 (13.5%)** missing: 3	**12 (22.6)** missing: 1	3 (3.7) missing: 1

*Note*: Bold–Significant only if *p* < 0.01 between controls and the examined diagnostic group.

^a^ No concomitant inflammatory condition.

The most prevalent diagnosis among our patients was NMSC, including AK, *N* = 81, of which 72 (28.2%) had NMSC/AK as a primary diagnosis. Non‐melanoma skin cancer/AK was followed by psoriasis, *N* = 54, with 47 (18.4%) as a primary diagnosis. Next were patients with metabolic or systemic disease, 23 (9%), and adult atopic dermatitis, 15 (5.9%) (Table S1).

### 3.2 Perceived stress using the PSS‐10

Considerable levels of stress (moderate or high, PSS > 13) were reported by significantly more patients than controls (59.8% and 40% respectively, *p* < 0.01), in 66% of patients with inflammatory disease (*p* < 0.001), and in 46.5% of patients with a non‐inflammatory disease (non‐significant, *p* = 0.2 compared to controls).

In Table 2 we present psychosocial characteristics (PSS, PHQ‐2, GAD‐2, EQ‐VAS, possibilities for education, employment and economic difficulties) of patients compared with controls, and show significant differences at the *p* < 0.01 level for inflammatory diseases compared with controls.

**TABLE 2:** Health related characteristics for patients and controls

Population	Controls *N* = 148	All patients *N* = 255	Inflammatory *N* = 173	Psoriasis *N* = 54	Non‐inflam‐matory^a^ *N* = 82
PSS^b^ mean (SD)	12.9 (6.4) missing: 8	**15.5 (7.4)** missing: 31	**16.6 (7.4)** missing: 20	**16.4 (6.8)** missing: 6	13 (6.7) missing: 11
PSS > 13^c^ *N* (%)	56 (40%) missing: 8	**134 (59.8)** missing: 31	**101 (66%)** missing: 20	**32 (66.7)** missing: 6	33 (46.5%) missing: 11
Stressful event last 6 months ‘yes’ *N* (%)	29 (20.4) missing: 6	73 (30.7) missing: 17	48 (29.4) missing: 10	20 (37.7) missing: 1	25 (33.3) missing: 7
EQ‐VAS mean (SD)	83.3 (12) missing: 1	**67.9 (22.2)** missing: 10	**63.1 (22.6%)** missing: 7	**61.7 (20.5)** missing: 1	**78 (17.6)** missing: 3
Comorbidity ‘yes’ *N* (%)	36 (24.8%) missing: 3	**179 (70.2%)** missing: 0	**116 (67.1%)** missing: 0	**35 (64.8%)** missing: 0	**63 (76.8%)** missing: 0
PHQ2^d^ ≥ 3 *N* (%)	12 (8.2%) missing: 2	**46 (18.7%)** missing: 11	**41 (24.6%)** missing: 6	**13 (25%)** missing: 2	5 (6.3%) missing: 3
GAD2^e^ ≥ 3 *N* (%)	20 (13.5%) missing: 0	**63 (26%)** missing: 13	**51 (20.7%)** missing: 7	**18 (35.3%)** missing: 3	12 (15.8%) missing: 6

*Note*: Bold–Significant only if *p* < 0.01 between controls and the examined diagnostic group.

^a^ No concomitant inflammatory skin condition.

^b^ **PSS**: Perceived Stress Scale.

^c^ **PSS>13** denoting considerable perceived stress (moderate to high).

^d^ **PHQ2** (Patient Health Questionnaire).

^e^ **GAD2** (General Anxiety Disorder Assessment). Values of three or higher indicate that the individual needs to be screened for depression or anxiety.

For psoriasis, sex distribution was similar to controls. Age, education possibilities, employment and considerable economic difficulties, as well as health‐related factors (PSS, EQ‐VAS, depression (PHQ‐2) and anxiety (GAD‐2)) differed significantly from the controls (*p* < 0.01).

The ‘Non‐inflammatory’ group showed significant differences compared with controls only for age, EQ‐VAS and comorbidities. We did calculations separately for only benign non‐inflammatory conditions, separately for only cancers and melanoma and pooled benign, cancers and melanoma. The results remained similar and we chose to examine both benign and non‐benign non‐inflammatory conditions pooled (data not presented for the separate calculations).

The risk of having moderate to high perceived stress was more than doubled in dermatological patients compared to controls when controlling for age and sex, having a stressful life event and comorbidity (OR and 95% Confidence Interval = 2.5 (1.4–4.3)). For inflammatory skin conditions, the risk was tripled (OR (95% CI) = 3.1 (1.7–5.6)), as was for psoriasis 3.1 (1.4–7.2). On the contrary, non‐inflammatory skin conditions did not show any higher risk for perceived stress compared with controls (Table 3).

**TABLE 3:** Perceived stress ‘moderate to high’ defined as PSS > 13 using the Perceived Stress Scale (PSS‐10), in patients compared with controls adjusted for age, gender, experienced significant stressful life event during the last 6 months and having a comorbidity

Controls *N* valid = 132	*N* valid	OR (95% CI)
All patients *N* = 255	213	2.5 (1.4–4.3)
Inflammatory^a^ *N* = 173	146	3.1 (1.7–5.6)
Psoriasis^a^ *N* = 54	47	3.1 (1.4–7.2)
Non‐inflammatory^b^ *N* = 82	67	1.3 (0.6–3.3)

*Note*: Presented as Odds Ratio (OR) with 95% Confidence Interval (95% CI). Participants with a missing answer for PSS were not included in the calculations.

^a^ Either primary and/or secondary diagnosis.

^b^ No concomitant inflammatory skin condition.

## 4 DISCUSSION

Stress and skin diseases often co‐occur.^7^, ^8^, ^9^, ^15^ Compared to controls, patients with skin disease in our study were 2.5 times more likely to report perceived stress. For inflammatory skin conditions and for psoriasis the risk was tripled, but there were no significant differences between controls and patients with non‐inflammatory skin conditions.

Comparing diagnostic groups with controls by sociodemographics and health‐related factors allowed us to see if there were any significant differences between the abovementioned diagnostic groups and controls, while adjusting for important confounders gives more sound data for the risk of perceiving stress in dermatology.

Concern with body image is a common reaction to skin cancer and its treatment, which may cause visible, unsightly scars.^41^ These concerns and fright for malignancy may cause stress even without an inflammatory component. It was therefore interesting that in spite of those factors, the risk for perceived stress in non‐inflammatory skin diseases remained insignificant compared with controls. A study by Misery et al. similarly shows no significant differences in stress between patients with naevi and controls.^9^

Stress is linked to various health outcomes, the most explored being diabetes, cardiovascular disease, asthma, cancer and rheumatoid arthritis^2^, ^37^ and leads to reduced health in several aspects, with productivity losses for the individual and for society, as well as a negative impact on treatment.^24^ The same can be assumed for chronic skin diseases as emotional problems and distress in inflammatory skin conditions are high,^7^, ^28^, ^42^ in accord with our results. Dermatological studies have examined stress in patients with psoriasis,^9^, ^19^, ^22^, ^23^, ^27^, ^42^ vitiligo,^8^ atopic eczema,^9^, ^42^ acne,^9^, ^25^, ^42^ urticaria^43^ and seborrhoeic dermatitis,^42^ confirming higher perceived stress in inflammatory skin disease.

Less knowledge exists on a larger range of skin conditions and in an outpatient setting, except for one study in older outpatients.^10^ Sampogna et al. likewise report that older patients with psoriasis experienced higher distress,^23^ where multiple health issues were more prevalent. We adjusted for age and comorbidities to control for these factors.

In another study^22^ PSS scores in psoriasis patients were positively correlated with the number of life events. We adjusted for experienced stressful events, precisely to control for any stressful event that may not be related to the skin condition.

Most studies evaluate stress and disease exacerbation by retrospective recall leading to recall bias. An exception is a study that used a diary technique to prospectively assess the skin disease symptoms, depressive symptoms, and interpersonal stress in atopic eczema.^44^ The conclusion was that atopic eczema causes depression and not vice versa. Significant correlation between exam stress and students' acne severity was seen in a small prospective study.^25^ These studies may suggest a directionality between stress and exacerbation of skin disease. In the present study we focussed on perceived stress and inflammation, but adjust for recently experienced stress. A bidirectional, self‐perpetuating process is very likely.

Findings on the relationship of depression and anxiety leading to higher perceived stress, and thereby relapse of an inflammatory skin disease may have considerable clinical implications, such as including psychological interventions in the management of skin disease.^11^ Gupta et al. suggest that when assessing a patient with psoriasis, the clinician should specifically inquire whether the patient's psoriasis is stress reactive and if so, the psoriasis should be treated with medications that achieve remission faster.^6^

Given the prevalence of skin diseases and the burden of suffering associated with them, examining factors that may lead to exacerbation are of importance. Stress reduction techniques and psychological interventions have been proposed as adjunctive treatments in skin disease^6^ and further trials for evaluating efficacy of such techniques are indicated.

Knowledge on associations between stress and different skin conditions may help in resource allocations and planning of dermatological consultations with faster referral if a diagnosis is known to cause higher perceived stress.

Currently, skin cancers are prioritised in Norway, and if cancer is suspected, waiting time is very short.^45^ Acute and exacerbated inflammatory skin conditions, likewise, are prioritised, but a large amount of patients with non‐exacerbated, or less serious inflammatory skin conditions, including psoriasis, may need to wait several months for a consultation.^45^ Patients with skin conditions may experience higher stress, also because of the distress of not receiving timely help if the condition is not highly prioritised. Psychological factors such as coping mechanisms, depression and personality traits are related to stress.^19^ We hope that our study will heighten the awareness around the need of prioritising those conditions even when non‐malignant or not exacerbated.

The results we present are valid for a Norwegian outpatient population before the Covid‐19 pandemic and show perceived stress in a population where only individual, personal factors, without the influence of a pandemic have played a role. Comparing pre and post pandemic data may be interesting.

### 4.1 Strengths

We describe the risk of experiencing stress in patients with inflammatory dermatoses and non‐inflammatory dermatoses compared with controls and adjust for several factors. We chose to exclude any concurrent inflammatory skin condition when analysing patients with non‐inflammatory dermatoses to attain pure results for non‐inflammatory dermatoses. We therefore look at the relationship between stress and having an inflammatory skin condition or not. There is very little previous research on stress in patients with non‐inflammatory skin conditions.

### 4.2 Limitations

This is a single centre cross‐sectional study, which only provides odds of perceiving stress in patients with inflammatory and non‐inflammatory skin diseases. Due to its cross sectional design, it is not possible to draw any conclusions regarding causality and directionality. Results are not generalisable to other populations. We only present results acquired at the time of the dermatological consultation.

## 5 CONCLUSIONS

Perceived stress is considerably higher in patients with inflammatory dermatoses compared to controls, but not in patients with non‐inflammatory skin conditions. Bidirectionality through a self‐perpetuating mechanism is suggested by biomechanisms previously explored, but the design of our study does not allow for conclusions on directionality. Performing longitudinal prospective randomized control studies may provide evidence of direction of causality and further studies examining this are recommended. We hope our results can serve to motivate health authorities in Norway (or elsewhere) to prioritise the treatment of patients with benign inflammatory skin conditions at least to the level of patients with skin cancer.

## AUTHOR CONTRIBUTIONS

**Flora Balieva**: Conceptualization (lead); Data curation (Supporting); Formal analysis (equal); Funding acquisition (lead); Investigation (lead); Methodology (equal); Project administration (equal); Resources (equal); Software (Supporting); Supervision (equal); Validation (lead); Visualization (lead); Writing – original draft (lead); Writing – review & editing (lead). **Christina Schut**: Conceptualization (lead); Methodology (lead); Project administration (lead); Supervision (lead); Validation (equal); Writing – original draft (Supporting); Writing – review & editing (Supporting). **Jörg Kupfer**: Conceptualization (lead); Data curation (lead); Formal analysis (lead); Methodology (lead); Project administration (lead); Supervision (equal); Validation (Supporting); Writing – review & editing (Supporting). **Lars Lien**: Conceptualization (equal); Methodology (equal); Supervision (equal); Validation (equal); Writing – original draft (Supporting); Writing – review & editing (Supporting). **Laurent Misery**: Supervision (equal); Validation (equal); Writing – review & editing (Supporting). **Francesca Sampogna**: Formal analysis (equal); Validation (equal); Writing – review & editing (equal). **Love von Euler**: Data curation (Supporting); Investigation (Supporting); Validation (Supporting); Writing – original draft (Supporting); Writing – review & editing (Supporting). **Florence J. Dalgard**: Conceptualization (lead); Data curation (Supporting); Investigation (equal); Methodology (lead); Supervision (equal); Validation (equal); Writing – review & editing (Supporting).

## CONFLICT OF INTEREST

No conflicts of interest to declare.

## ETHICS STATEMENT

The Institutional Review Board at Stavanger University Hospital has approved the study. ID 702/2017.

## Supporting information

Supplementary Material

## Data Availability

The data that support the findings of this study are available on request from the corresponding author. The data are not publicly available due to privacy or ethical restrictions.
