# Supplementary material for: Perceived stress in patients with inflammatory and non‐inflammatory skin conditions. An observational controlled study among 255 Norwegian dermatological outpatients
Source: Skin Health Dis. 2022 Aug 31;2(4):e162. doi: 10.1002/ski2.162 (PMC9720195; doi:10.1002/ski2.162)
Supplement: Supplementary file 1 — Supplementary Material [file SKI2-2-e162-s001.docx]

**Table S1. Distribution of diagnoses and diagnostic category. Presented by descending frequency.**

| **Dermatological diagnosis (ICD-10)** | **N (%)** | **Grouping in diagnostic category** |
| --- | --- | --- |
| Non-melanoma skin cancer and actinic keratosis (C44.-, L57.0) | 72 (28.2) | Non-inflammatory |
| Psoriasis (L40.-) | 47 (18.4) | Psoriasis (Inflammatory) |
| Systemic and metabolic disease (D60–89, E, G, I73, I77, I78, L88, R20, Z94.-) | 23 (9.0) | Inflammatory |
| Adult Atopic Dermatitis (L20.-) | 15 (5.9) | Inflammatory |
| Hyperhidrosis (R61.-) | 12 (4.7) | Inflammatory |
| Naevi (D22.-) | 8 (3.1) | Non-inflammatory |
| Skin malformations (epidermal nevi, vascular malformations, atrohy) (L90, Q80, Q82) | 8 (3.1) | Non-inflammatory |
| Rosacea, perioral dermatitis (L71.-) | 8 (3.1) | Inflammatory |
| Hidradenitis suppurativa (L73.2) | 7 (2.7) | Inflammatory |
| Pruritus (L29.-) | 7 (2.7) | Inflammatory |
| Acne (L70.-) | 5 (2.0) | Inflammatory |
| Malignant Melanoma/in situ (C43.- D03.-) | 5 (2.0) | Non-inflammatory |
| Urticaria (L50.) | 5 (2.0) | Inflammatory |
| Scaly conditions (L41 – L45) | 5 (2.0) | Inflammatory |
| Hand eczema* | 3 (1.2) | Inflammatory |
| Benign Tumours (D17, 18, 21, 23, 24.3, 25.5) | 3 (1.2) | Non-inflammatory |
| Allergic, hypersensitivity reactions (L23, 27, J) | 3 (1.2) | Inflammatory |
| Other dermatitis/eczemas (L22, L24, L25, L30) | 3 (1.2) | Inflammatory |
| Seborrheic dermatitis (L21) | 3 (1.2) | Inflammatory |
| Prurigo (L28.-) | 2 (0.8) | Inflammatory |
| Connective tissue disease (L93 – L95) | 2 (0.8) | Inflammatory |
| Other (balanitis, oral apthae) N48.1, K12.0 | 2 (0.8) | Inflammatory |
| Infections (Pityriasis versicolor, Herpes simplex) | 2 (0.8) | Inflammatory |
| Vesiculo-bullous disorders (L10–L13, O26.4) | 1 (0.4) | Inflammatory |
| Other alopecias (L64 – L65) | 1 (0.4) | Inflammatory |
| Venous insufficiency (I80, I83, L97) | 1 (0.4) | Inflammatory |
| Vitiligo, disturbance of pigmentation (L80-81) | 1 (0.4) | Inflammatory |
| Psychodermatological (Dermatitis facticia, L98.1) | 1 (0.4) | Inflammatory |
| Alopecia areata (L63) | 0 | Inflammatory |
| **Total** | **255** | **All (Inflammatory + Non-inflammatory)** |
| *Hand eczema: If localization is only on the hands (L20.-; L23. – L25.-; L30.-; L27.-)  A more detailed ICD-10 list can be found at: Schut, C., et al., Body dysmorphia in common skin diseases: Results of an observational, cross-sectional multi-centre study among dermatological out-patients in 17 European countries. Br J Dermatol, 2022 [doi: 10.1111/bjd.21021](https://pubmed.ncbi.nlm.nih.gov/35041211/) | | |
